# Midgut membrane protein BmSUH facilitates *Bombyx mori* nucleopolyhedrovirus oral infection

**DOI:** 10.1371/journal.ppat.1010938

**Published:** 2022-11-16

**Authors:** Yanting Liang, Weifan Xu, Yanyan Zhou, Yun Gao, Huan Tian, Xiaofeng Wu, Yusong Xu, Huabing Wang

**Affiliations:** College of Animal Sciences, Zhejiang University, Hangzhou, China; University of Wisconsin-Madison, UNITED STATES

## Abstract

Baculoviruses are virulent pathogens that infect a wide range of insects. They initiate infections via specific interactions between the structural proteins on the envelopes of occlusion-derived virions (ODVs) and the midgut cell surface receptors in hosts. However, host factors that are hijacked by baculoviruses for efficient infection remain largely unknown. In this study, we identified a membrane-associated protein sucrose hydrolase (BmSUH) as an ODV binding factor during *Bombyx mori* nucleopolyhedrovirus (BmNPV) primary infection. BmSUH was specifically expressed in the midgut microvilli where the ODV-midgut fusion happened. Knockout of BmSUH by CRISPR/Cas9 resulted in a significantly higher survival rate after BmNPV orally infection. Liquid chromatography-tandem mass spectrometry analysis and co-immunoprecipitation analysis demonstrated that PIF protein complex required for ODV binding could interact with BmSUH. Furthermore, fluorescence dequenching assay showed that the amount of ODV binding and fusion to the midgut decreased in *BmSUH* mutants compared to wild-type silkworm, suggesting the role of BmSUH as an ODV binding factor that mediates the ODV entry process. Based on a multilevel survey, the data showed that BmSUH acted as a host factor that facilitates BmNPV oral infection. More generally, this study indicated that disrupting essential protein-protein interactions required for baculovirus efficient entry may be broadly applicable to against viral infection.

## Author summary

*Baculoviridae* is a large family of pathogens that infect insects and frequently cause fatal diseases. *Bombyx mori* nucleopolyhedrovirus (BmNPV) is a major threat to the sericulture industry. Although we have learned a lot about baculoviruses over the past several decades, the detailed interaction patterns between host proteins and viral proteins that lead to infection remain underexplored. Here, we determined that BmSUH, a midgut microvilli protein, was required for the efficient oral infection of BmNPV. Our research suggests that BmSUH mediates the entry of occlusion-derived virions into the midgut epithelia by interacting with *per os* infectivity factors. According to the findings, inhibition of viral binding to host cells is an attractive strategy to prevent infection. This study provides an approach for preventing BmNPV infection through developing genetic resistance to viruses by using CRISPR/Cas9 system to abolish the host factors that are essential for viral entry.

## Introduction

Baculoviruses (family: *Baculoviridae*) are pathogenic viruses that infect invertebrates and represent a large group of enveloped, rod-shaped viruses with double-stranded DNA [1,2]. One hallmark of the baculovirus infection cycle is the generation of two morphologically and functionally distinct progeny phenotypes: the budded virus (BV) and the occlusion-derived virus (ODV). ODVs are enveloped virus particles embedded in a protein crystal known as occlusion body (OB)[3]. Once released from OBs under the alkaline conditions of the insect midgut, ODVs enter the epithelial cells of the midgut by binding and fusion with the membrane and initiate primary infection [4,5]. Due to the complexity of *in vivo* studies and the lack of midgut cell lines supporting ODV entry, the detailed entry mechanisms directing primary infection processes have not been addressed till date [6,7]. A group of ODV envelope proteins, termed *per os* infectivity factors (PIFs) have been found to be essential for the oral infection process as the deletion of any single PIF would lead to invalid oral infection [8]. Except PIF5, the other 8 PIFs and the homologue of AcmNPV PIF9, Bm91 act in a PIF complex [9].

Given the probable saturation of ODV binding to midgut epithelial cells, a competitive experiment suggested that particular cellular receptor(s) interacting with PIF0 were present in the midgut epithelia [10]. So far, several microvillar proteins of the midgut have been found to interact with PIF proteins. For instance, PIF0 (P74) from *Helicoverpa armigera* nucleopolyhedrovirus (HearNPV) and *Autographa californica* multicapsid nucleopolyhedrovirus (AcMNPV) binds to unknown 30 kDa and 35 kDa proteins from their hosts’ brush border membrane vesicles (BBMV) [11,12]. A 97 kDa unknown protein from BBMV was also found to bind to PIF5 in *Heliothis virescens* [13]. However, the nature of these binding factors or receptor(s) on midgut epithelial cells remains unknown [7]. *Bombyx mori* nucleopolyhedrovirus (BmNPV), which belongs to *Alpbabaculovirus* genus, is an exclusive pathogen of the silkworm, frequently causing grasserie disease and severe economic damage to sericulture production [14,15]. Inhibiting viral entrance into midgut cells is an attractive strategy to prevent BmNPV infection. It is of great significance to investigate the essential host factors in regulating the establishment of infection.

α-Glucosidase (EC 3.2.1.20) is a large family of glucoside hydrolases, whose main function is to hydrolyze glucoside bonds and release glucose as a product [16,17]. In recent years, several studies have demonstrated that α-glucosidases are functionally diverse. Previous studies reported the membrane-bond α-glucosidase from midgut microvilli aided pathogen invasion [18]. For instance, Culex quinquefasciatus maltase 1, Agm3 and Cpm1 in mosquito served as receptors of *Bacillus sphaericus* binary toxin, Bta11975 and *Bemisia tabaci α-*glucosidase 1 promoted the transmission of tomato chlorosis virus [19–22]. In mammals, α-glucosidases have been widely reported in facilitating the efficient infection of multiple envelope viruses, including *Dengue virus*, *Hepatitis C virus*, *Hepatitis B virus*, and *human immunodeficiency virus* [23–27]. Previous studies found that a membrane-bond α-glucosidase, sucrose hydrolase (BmSUH, also known as maltase A1) showed significant expression level changes after BmNPV infection [28,29]. In addition, comparative transcriptome analysis of BmNPV-susceptible and -resistant silkworm strains showed that BmSUH exhibited novel alternative splicing when the BmNPV-susceptible silkworm strain was exposed to BmNPV [30]. These data suggest that BmSUH may be involved in BmNPV infection process. SUH was highly expressed in the midgut of lepidopteran insects including butterflies (*Papilio xuthus*) and moth (*B*. *mori*, *Samia cynthia ricini*, and *Trilocha varians*) [31–33]. Previous studies have focused on its function as a sucrose hydrolase, but the extent to which BmSUH is involved in BmNPV infection process remains unexplored.

In this study, we performed a series of functional studies to characterize the role of BmSUH in BmNPV infection. We applied the CRISPR/Cas9 system to directly disrupt BmSUH and investigated its potential function during the BmNPV infection process. The liquid chromatography-mass spectrometry (LC-MS/MS) and co-immunoprecipitation (Co-IP) were employed to investigate which viral proteins interacted with BmSUH. Fluorescence-dequenching assay was used to investigate if BmSUH mediates ODV binding and fusion. The data found that BmSUH facilitated the establishment of an efficient infection in *B*. *mori* midgut via interacting with one or more proteins in the PIF complex. These findings suggest that BmSUH acts as a host cofactor for efficient oral infection of BmNPV.

## Results

### BmSUH is specifically expressed in the midgut at the larval stage and localized in the plasma membrane

**Fig 1:**
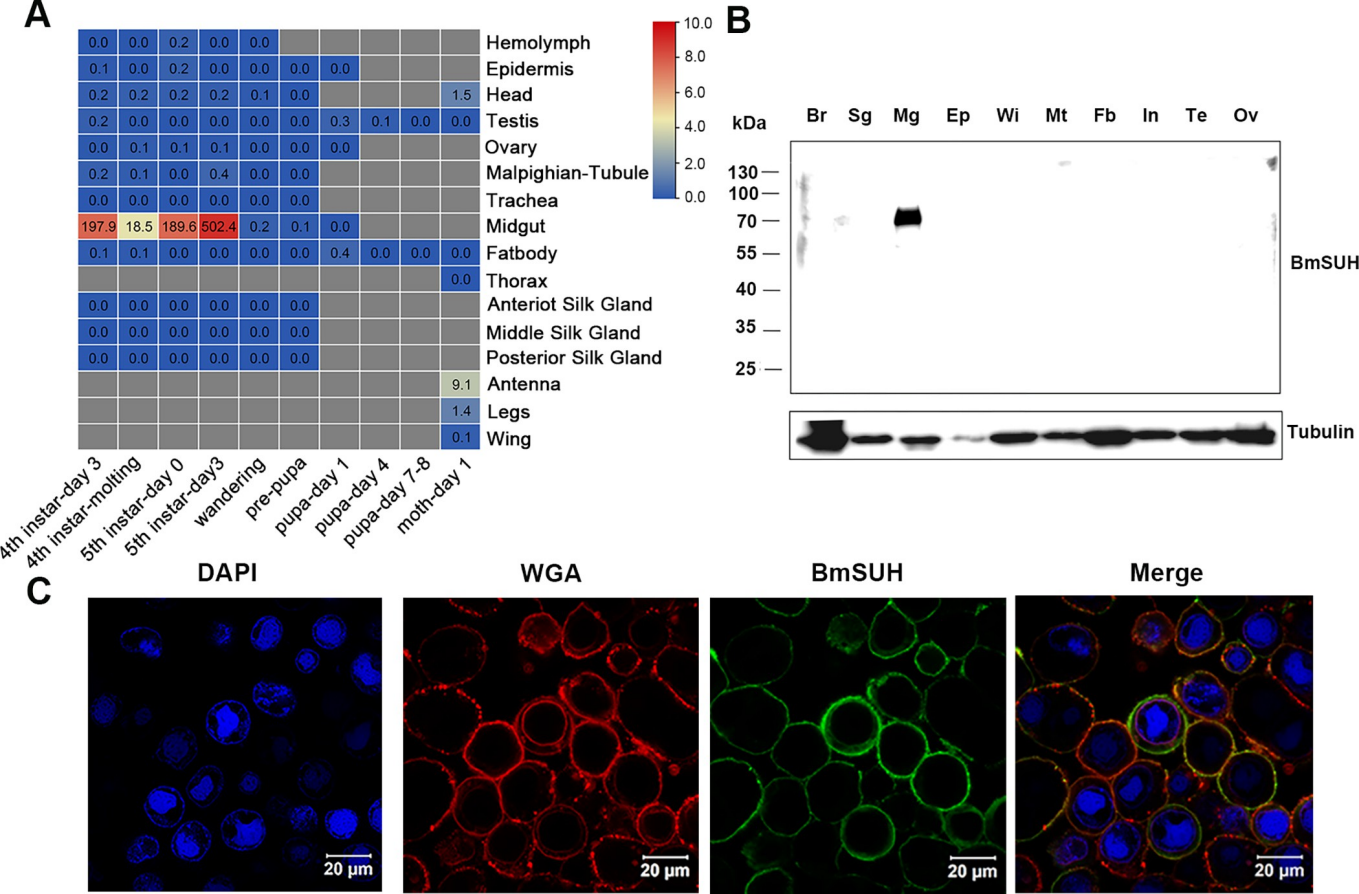
BmSUH is a membrane-associated midgut protein. (A) Expression patterns of *BmSUH* mRNA in *B*. *mori* expression data from SilkDB 3.0 (https://silkdb.bioinfotoolkits.net). (B) Expression profiles of BmSUH in the third day of fifth instar larvae (L5D3). Immunoblot analysis were performed using antibody against BmSUH and α-Tubulin as a loading control. Protein samples were isolated from brain (Br), silk gland (Sg), midgut (Mg), epidermis (EP), wing disc (Wi), Malpighian tube (Mt), fat body (Fb), integument (In), testis (Te), and ovary (Ov). (C) Subcellular localization of BmSUH in BmN cells. BmSUH was expressed in BmN cells by transfection. Antibody specific to BmSUH was used for immunofluorescence and green fluorescence (FITC) was observed as a marker. The cytoplasmic membrane was stained red using WGA-AF 594 and nuclei were visualized using 4’,6’-diamidino-2-phenylindole (DAPI, blue) counterstain.

To determine the temporal expression profiles of *BmSUH* across tissues and developmental stages in *B*. *mori*, we retrieved the data from SilkDB 3.0 [34]. The RNA-seq profiling revealed that the expression of *BmSUH* was specifically detected in larval midgut and mainly during the feeding stage (**Fig 1A**). We next investigated the expression and localization of BmSUH protein on the third day of the fifth instar (L5D3) larvae by immunoblot analysis. The result showed that BmSUH was exclusively expressed in the midgut (**Fig 1B**), which is consistent with *BmSUH* transcript levels (**Fig 1A**)[31]. A transmembrane domain of BmSUH was predicted by the TMHMM and SOSUI [31,35]. To further investigate the subcellular localization of BmSUH, we constructed PIZ-BmSUH plasmid that encoded transiently expressed BmSUH protein. BmN cells were transfected with the PIZ-BmSUH plasmid and the distribution of BmSUH was determined by immunofluorescence straining using anti-BmSUH antibody. As shown in **Fig 1C**, BmSUH was stained positively in the plasma membrane.

### Construction of *BmSUH* deletion mutants

**Fig 2:**
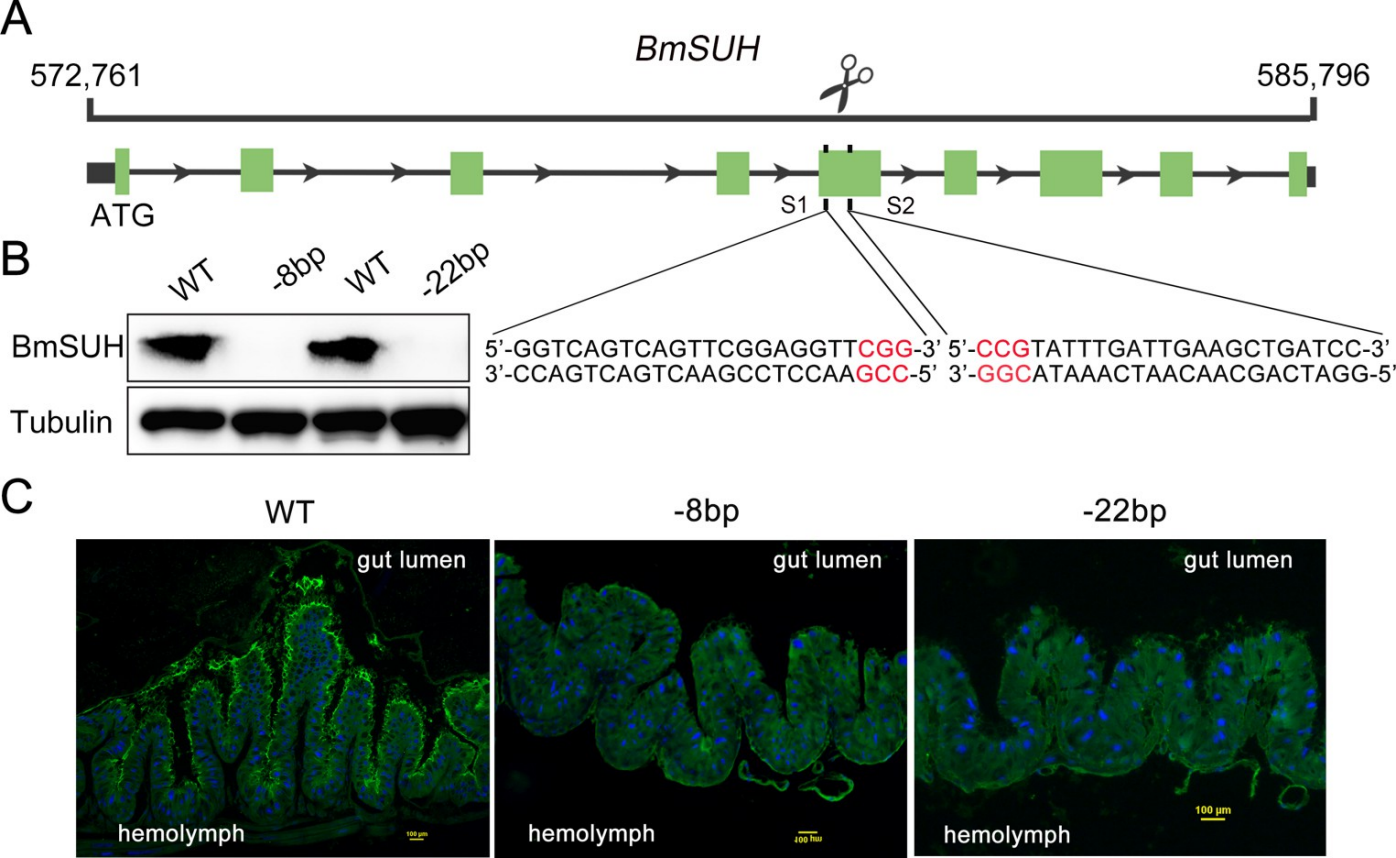
Knockout of *BmSUH* in *B*. *mori*. (A) Schematic description of the *BmSUH* gene and the sgRNA target site. Black and green squares represent the noncoding (UTRs) and coding parts of the transcript, respectively. The sgRNA targeting sites, S1 and S2, are located on the forward strand and reverse strand of the fifth exon, respectively. The sgRNA sequences are depicted in black and the corresponding PAM sequence is marked in red. The scissors image was obtained from the “Icon Library” of iSlide application under a CC0 1.0 Universal license. Copyright: https://en.islide.cc/copyright-notice. (B) Immunoblot validation of two types of *BmSUH* mutants. (C) Immunofluorescence staining of BmSUH (green) on midgut tissue sections of wild-type and *BmSUH* deficient silkworms. Silkworm specimens were obtained from L5D3. DAPI (blue) served as a nuclear dye. WT: wild-type; -8bp and -22bp: two *BmSUH* mutant lines.

To further investigate the function of *BmSUH*, we constructed *BmSUH*-knockout mutants using the CRISPR-Cas9 system. Two small guide RNAs (sgRNA) were designed to target the fifth coding-exon within the *BmSUH* gene (**Fig 2A**). Only the sgRNA site 1 was highly efficient. Finally, two independent types of genomic deletions were obtained, where 8 bp or 22 bp were deleted compared with the wild-type (WT) (**S1 Fig**). The mutation caused a complete loss of BmSUH protein expression (**Fig 2B**). *B*. *mori* midgut consists of a monolayered epithelium essentially formed by columnar and goblet cells. To precisely define the localization of BmSUH, we performed immunohistochemical staining in the midgut of silkworms. The strong signal was concentrated on apical microvilli of columnar cells, showing that BmSUH is a membrane-associated protein located in the midgut microvilli (**Fig 2C**). No signal was detected in two types of *BmSUH* mutants, further demonstrating a complete loss of BmSUH protein expression in these two mutants (**Fig 2C**).

Two *BmSUH* homozygous mutants displayed a nonlethal phenotype and produced heritable targeted mutations, showing that disruption of BmSUH had no deleterious effects on viability or fecundity (**S2 Fig**). As BmSUH is exclusively and abundantly expressed on microvilli, we observed midgut epithelial cells of WT and *BmSUH* mutants at L5D3. There is no visible significant difference in the structure of the midgut epithelium between WT and *BmSUH* mutants (**S3 Fig**). Compared to WT, *BmSUH* mutants prolonged the duration of the fifth instar by approximately 6 h. Since the characteristics of two homozygous individuals were identical, we chose the homozygous line whose genome lost 22 bp for experimental observations and described it as Δ*BmSUH*.

### *BmSUH* mutants exhibit higher resistance to BmNPV *per os* infection

**Fig 3:**
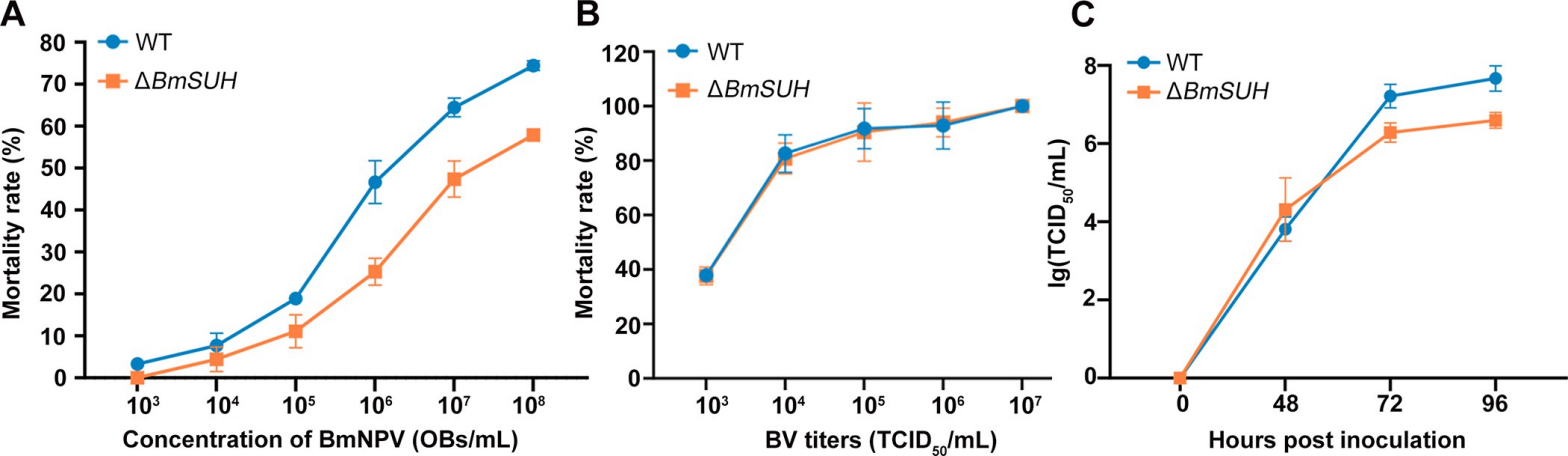
Larval bioassays. (A) The mortality analysis of fourth-instar WT and Δ*BmSUH* after being orally infected with the BmNPV at concentration of 1×10^3^, 1×10^4^, 1×10^5^, 1×10^6^, 1×10^7^ and 1×10^8^ OB/mL (n = 30). (B) The mortality of fifth-instar WT and Δ*BmSUH* after BV injection (n = 30). (C) The production of infectious BVs in the hemolymph of fourth-instar WT and Δ*BmSUH* after orally infected with 1×10^8^ OB/mL BmNPV. The hemolymph was taken at designed time points and the BV titers were determined by TCID_50_ endpoint dilution assays. Each data point was determined from the average of at least three independent experiments. The data represents the mean ± SEM.

Previous studies suggested that BmSUH may be involved in the BmNPV infection process [28–30]. To investigate the role of BmSUH in BmNPV infection *in vivo*, *per os* bioassays were conducted. Compared to control, the expression of BmSUH presented dynamic changes after BmNPV inoculation (**S4 Fig**). We next investigated whether *BmSUH* deletion had an effect on the infectivity of BmNPV in *B*. *mori* larvae. The median lethal concentration (LC50) of OBs was determined in 4th instar WT and Δ*BmSUH* after serial dilution of the viral stock. The LC50 determined in Δ*BmSUH* larvae was 3.7 × 10^7^ OB/mL, which was 13.45-fold of that determined in WT (**Table 1**). Correspondingly, the percent survival of Δ*BmSUH* was significantly higher than that of WT (**Fig 3A**). This result indicated that a higher viral dose was required to achieve lethality when *BmSUH* was deleted. We also assessed the median lethal time (LT50) by inoculating larvae with BmNPV at the concentration of 3.48 × 10^8^ OB/mL. The LT50 of *BmSUH* mutants was significantly longer than WT (**Table 2**).

**Table 1:** LC50 of fourth instar larvae after oral infection.

*B*. *mori*	LC50 (OBs/mL)	95% Fiducial limit	
		Lower	Upper
WT	2.75 ×10^6^	1.55× 10^6^	5.09×10^6^
Δ*BmSUH*	3.70×10^7^	1.98×10^7^	7.48×10^7^

**Table 2:** LT50 of fourth instar larvae after oral infection.

Larvae	LT50 (h)	95% Fiducial limit (h)	
		Lower	Upper
WT	96	91.568	100.432
Δ*BmSUH*	108	104.368	111.632

To determine whether the lower mortality of Δ*BmSUH* resulted from interactions of the virus with midgut cells or transfer of virus to cells in the hemocoel, we compared the virulence of BmNPV in control and mutant larvae by intrahaemocoelical injection of BV. The LD50 values of BV were 1.56 × 10^3^ and 1.92 × 10^3^ in WT and Δ*BmSUH*, respectively. These results showed no significant difference between WT and Δ*BmSUH* larvae (**Fig 3B and Table 3**). Taken together, these findings demonstrated that the absence of BmSUH enhanced the resistance of silkworm to BmNPV orally infection rather than BV infection, implying that BmSUH was mainly engaged in the midgut infective stage of primary infection.

**Table 3:** LD50 of fifth instar larvae after BV injection.

*B*. *mori*	LD50 (TCID_50_)	95% Fiducial limit	
		Lower	Upper
WT	1.56×10^3^	4.3×10^2^	3.87×10^3^
Δ*BmSUH*	1.92×10^3^	5.82×10^2^	4.46×10^3^

### BmSUH promoted the infection efficiency of BmNPV

To further explore the effect of BmSUH deletion on BV production, the hemolymph from silkworms that were orally infected with BmNPV was collected at the indicated time points. BmN cells were infected with the collected hemolymph and BV titers were determined by median tissue culture infectious dose (TCID_50_) endpoint dilution assay [36]. The data showed that the production of BV in WT was about 10–fold of that in *ΔBmSUH* at 72 and 96 hpi (**Fig 3C**).

**Fig 4:**
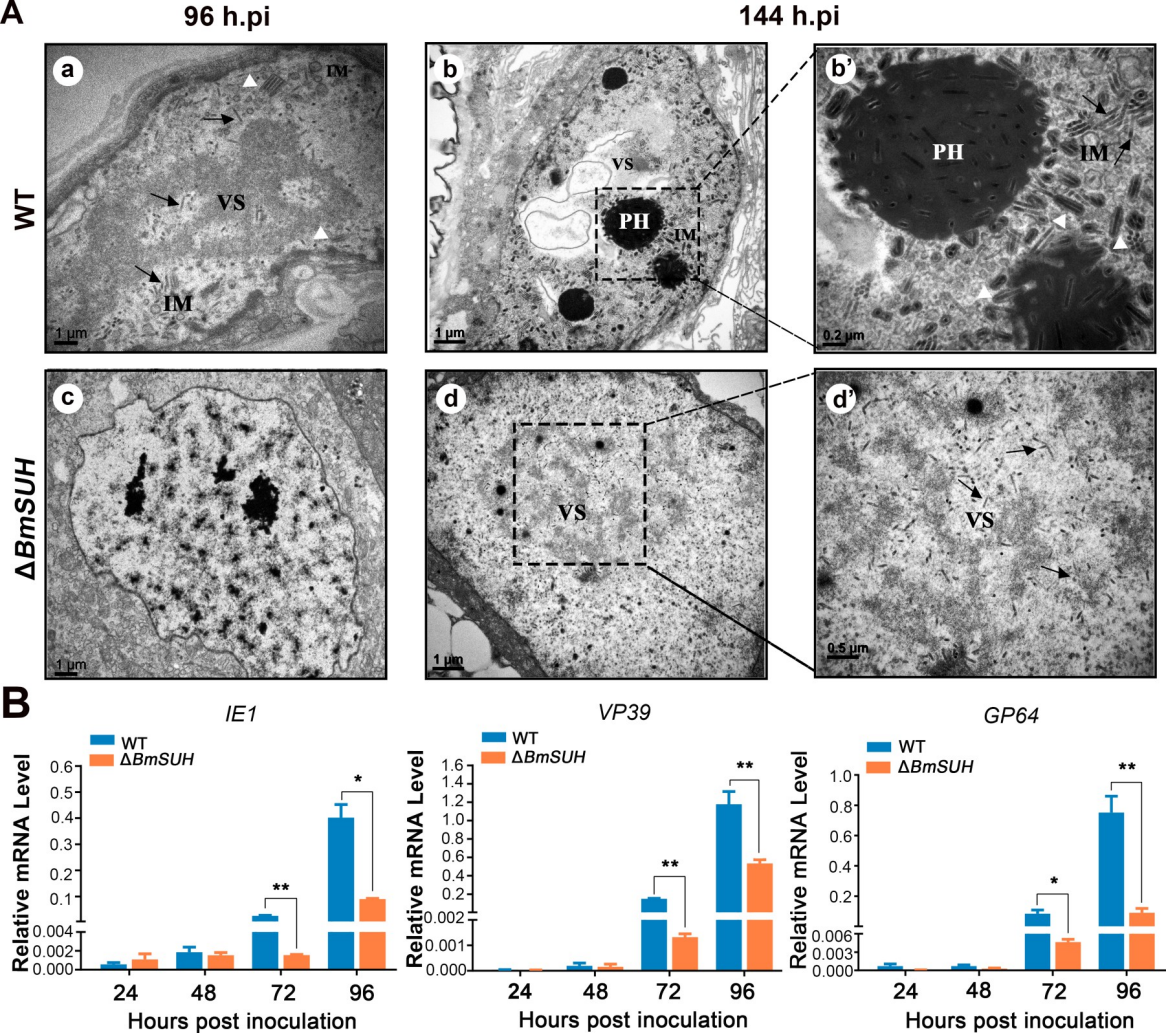
Interruption of BmSUH reduced the production of virions and viral gene expression. (A) TEM observations of virions in Δ*BmSUH* and WT larval midgut column epithelial cells. Electron-dense virogenic stroma (VS) and progeny nucleocapsids (black arrows) were observed both in the midgut of WT and Δ*BmSUH* larvae. The white triangles show intranuclear microvesicles (IM) and nucleocapsids associated with the membranous vesicular structures. PH, polyhedral. (B) Expression levels of different-phase viral genes, *IE1*, *GP64*, and *VP39* in WT or Δ*BmSUH* larvae midgut after inoculated orally with 10^6^ OBs (mean ± SEM). *p < 0.05 and **p < 0.01 by two-tailed Student *t* test.

To investigate whether the absence of *BmSUH* affects the formation, assembly, and transport of virions, the midgut samples derived from the orally infected larvae at the designated time points were excised for transmission electron microscopy (TEM) analysis. A typical BmNPV infection phenomenon was observed in WT (**Fig 4A**). At 96 hpi, a typical electron-dense virogenic stroma (VS) was observed in the nucleus of midgut cells, and the VS contained concentrated mature viral nucleocapsids. We found nucleocapsid envelopment into intranuclear microvesicles (IM) and embedding in polyhedra (PH) at 144 hpi (**Fig 4A**). In contrast, the obvious VS region and abundant rod-shaped nucleocapsids were not obviously observed in the midgut of the Δ*BmSUH* larvae until 144 hpi (**Fig 4A**). Moreover, the formation of the microvesicles and the ODV envelopment were hardly found in the midgut of Δ*BmSUH* (**Fig 4A**). To investigate the effect of *BmSUH* deletion on viral gene expression, we selected three major viral genes that correspond to three phases of BmNPV gene temporal expression pattern: *IE1* (an immediate-early gene), *GP64* (an early-and-late gene) and *VP39* (a late gene) [37,38]. Quantitative reverse-transcription PCR (RT-qPCR) analysis revealed a significant decreased mRNA expression level of *IE1*, *GP64*, and *VP39* in Δ*BmSUH* compared with that in WT at 72 and 96 hpi (**Fig 4B**). This result showed that the loss of BmSUH restrained the viral gene expression.

### Identification of BmSUH-associated proteins by Co-IP and LC-MS/MS

**Fig 5:**
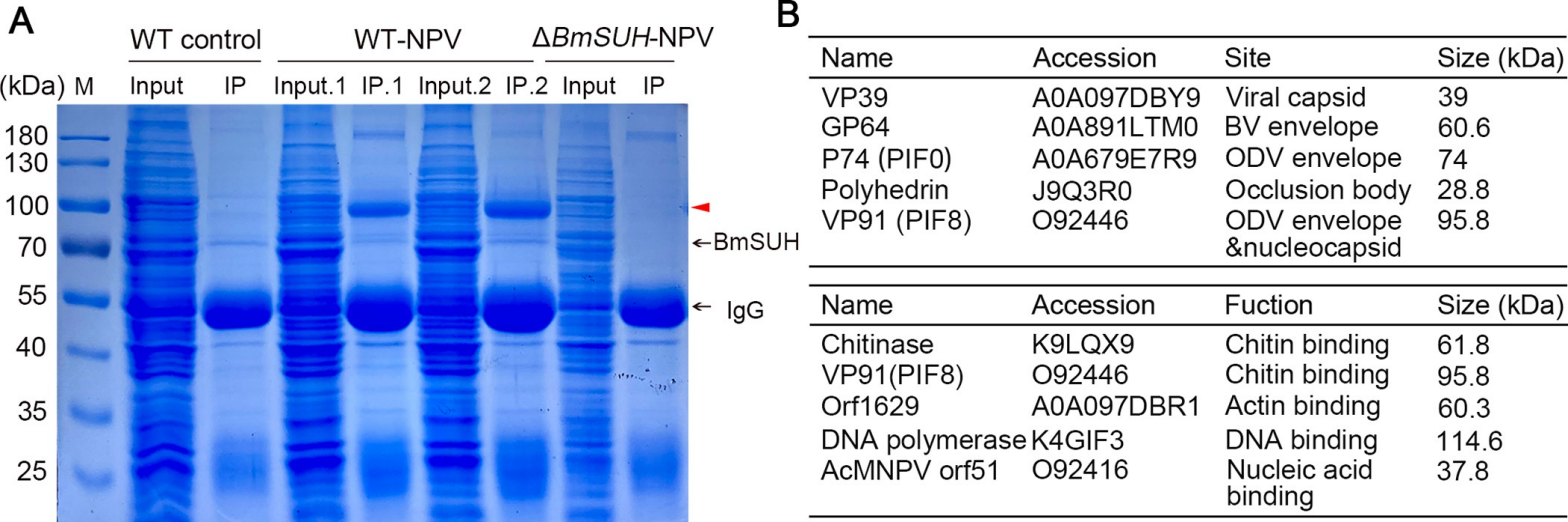
Identification of BmSUH interacting proteins by LC-MS/MS analysis of endogenous CO-IP products. (A) SDS-PAGE gel image of samples after endogenous BmSUH immunoprecipitation. Whole-cell lysates of midgut tissue were used for immunoprecipitation (IP) with anti-BmSUH. The red triangle represented the specific bands. (B) Viral proteins detected by LC-MS/MS in 70 kDa and 100 kDa gels. The structural proteins of BmNPV were shown in the upper graph and the proteins that belonged to the functional categories of “binding” in GO analysis were showed in the lower graph. IP.1: the IP sample at 72 hpi; IP.2: the IP sample at 96 hpi.

To investigate how *BmSUH* contributes to BmNPV orally infection, we investigated viral proteins that interact with BmSUH. Midgut proteins were extracted from uninfected WT, BmNPV-infected WT and BmNPV-infected Δ*BmSUH* larvae. Then endogenous BmSUH was immunoprecipitated from the midgut extracts using the BmSUH antibody. Compared to Δ*BmSUH* sample, two distinct bands of approximately 70 kDa and 100 kDa in WT samples were observed after BmNPV infection (**Fig 5A**). The 100 kDa band appeared only in BmNPV-infected WT samples, indicating that this band was a unique virus binding band (**Fig 5A**). To identify the viral and host proteins that were associated with BmSUH after BmNPV infection, the 70 kDa and 100 kDa bands were subjected to LC-MS/MS. A total of 631 BmSUH -associated host proteins were identified in the 100 kDa band. GO analysis revealed that the host proteins were mainly membrane associated, and predominantly enriched in molecule binding, such as small molecule binding (GO:0036094), nucleotide binding (GO:0000166), unfold-protein binding (GO:0051082) categories and so on (**S5 Fig**).

In addition, a total of 16 viral proteins were identified in 70 kDa and 100 kDa bands. Five of them were structural proteins of BmNPV and the others mainly belonged to the functional categories of “binding” (**Fig 5B and S1 Table**). The structural proteins included VP39, GP64, P74, Polyhedrin and VP91. The structural proteins form viral structures and are often required for initiating infection [39,40]. VP39 is thought to be the major capsid protein [41]. Polyhedrin is the major structural component of occlusion bodies [42]. GP64 is specific to BV envelopes, while VP91 (also known as PIF8) and P74 (also known as PIF0) are specific to ODV envelopes [7,43]. BmSUH associated with these major proteins of BmNPV in vivo, suggesting that BmSUH play an essential role during the midgut infection process.

### BmSUH interacts with PIF complex

**Fig 6:**
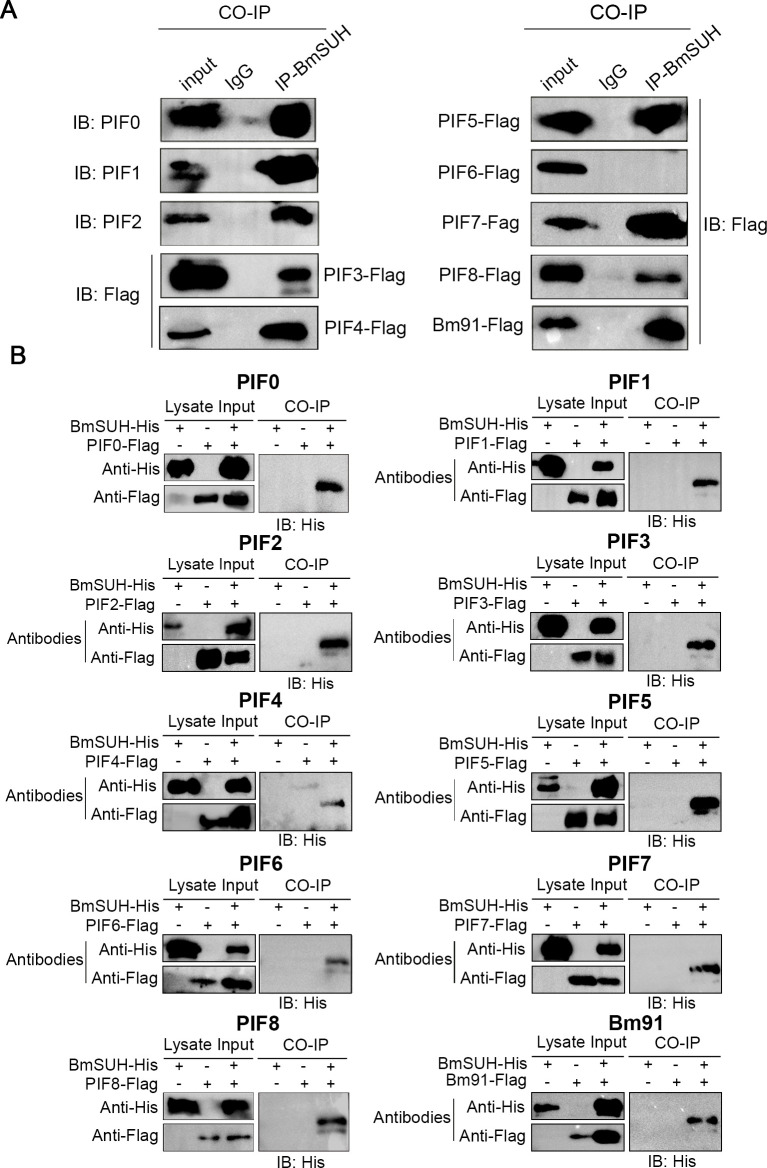
Co-immunoprecipitation analysis of interactions between different PIFs and BmSUH protein. (A) Co-IP analysis of BmSUH in NPV-infected BmN cells. BmN cells were infected with recombinant viruses expressing BmSUH-His or co-infected with BmSUH-His and PIFs-Flag. Immunoprecipitation (IP) was performed using anti-BmSUH or anti-IgG antibody followed by immunoblotting (IB) with anti-PIF0, anti-PIF1, anti-PIF2 or anti-Flag antibody individually. (B) BmN cells that were infected or co-infected with recombinant viruses expressing BmSUH-His or/and a Flag-tagged PIFs were subjected to anti-Flag. Inputs and eluates were analyzed by immunoblotting with anti-His.

PIFs are necessary for BmNPV orally infection but dispensable for BV infection [8]. Interestingly, two PIF proteins, PIF0 and PIF8, were detected in the LC-MS/MS result (**Fig 5B**). To confirm whether PIF0 and PIF8 interact with BmSUH, we performed Co-IP and reverse Co-IP assays in BmN cells as previously described [44]. The results showed that BmSUH could interact with PIF0 and PIF8 (**Fig 6**). Previous study found that PIF proteins act in a PIF complex, which contains PIF0, PIF1, PIF2, PIF3, PIF4, PIF6, PIF7, PIF8 and Bm91 [9]. BmSUH interacts with two components of the PIF complex, suggesting that it might be associated with the PIF complex. A series of Co-IP and reverse Co-IP assays were performed to explore whether BmSUH interacted with the PIF complex. The results showed that all components of the PIF complex had an interaction with BmSUH, suggesting that BmSUH interacts with the PIF complex (**Fig 6**). Although PIF5 was not a component of the PIF complex, it also had an interaction with BmSUH (**Fig 6**).

### Disruption of *BmSUH* decreased the binding and fusing efficiency of ODV with epithelial cell membranes

**Fig 7:**
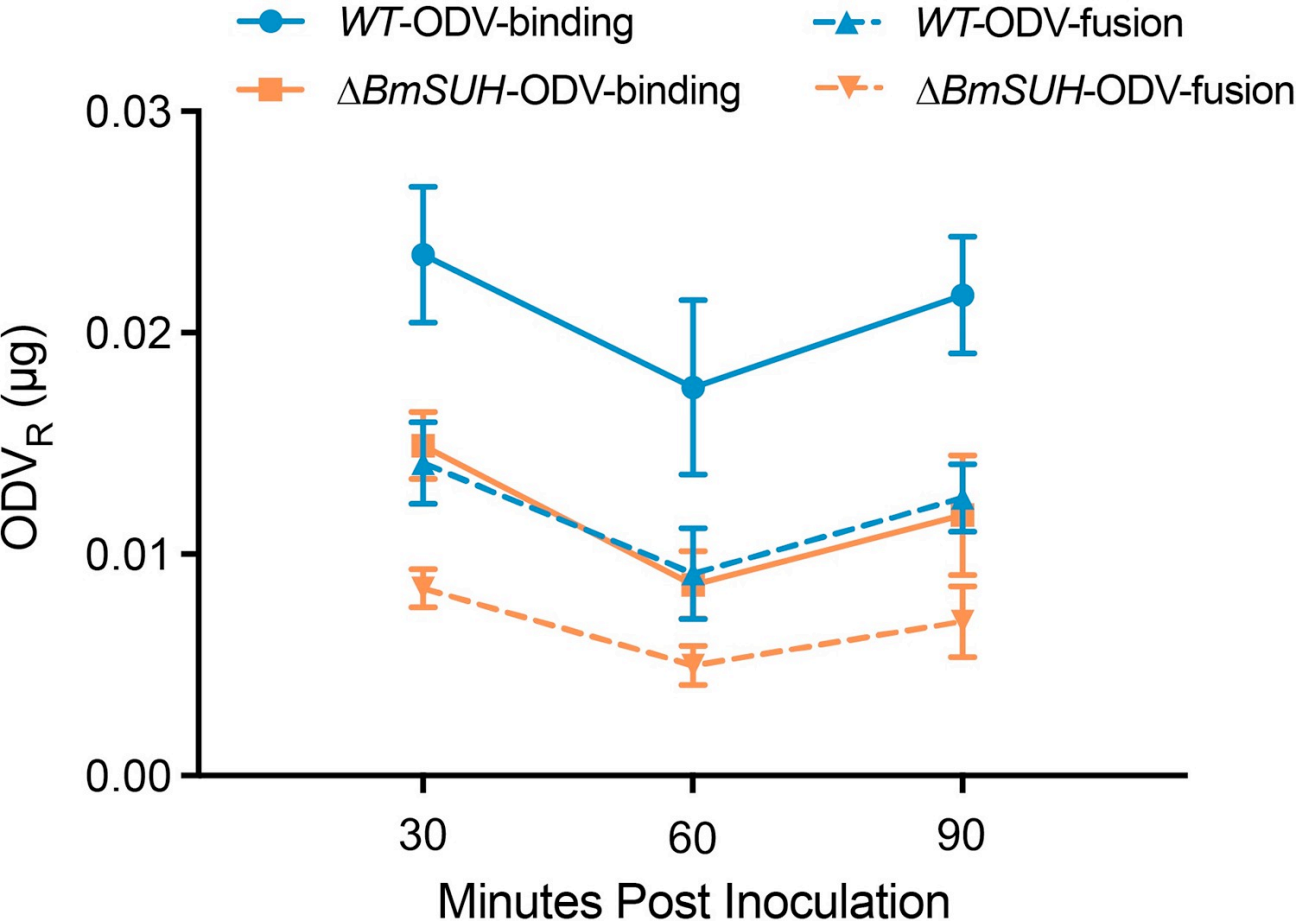
The absence of BmSUH affects the interaction of ODV with midgut epithelium cells. The amounts of labelled ODV_R_ bound (solid line) and fused (dotted line) to the midgut epithelia of fourth-instar WT and Δ*BmSUH* were detected after larvae feeding of 3 μg of labelled ODV_R_ from BmNPV and incubated with the designed time (n = 24, per time point). The data were analyzed using the GraphPad Prism 9 software. All data were shown as mean ± SEM.

Previous studies demonstrated that PIFs mediated the process of ODV binding and fusing to columnar cell microvilli of midgut [6,45]. To further investigate the role of BmSUH in the initial stages of baculovirus oral infection, we performed an octadecyl rhodamine B chloride (R-18) dequenching assay. This method relies upon the relief of fluorescence self-quenching of octadecyl rhodamine B chloride. We labeled ODV with a sufficiently high concentration of R18 (ODV_R_) to achieve self-quenching of the probe. Then we fed the ODV_R_ to fourth instar WT and Δ*BmSUH* larvae. When the ODV_R_ fused with the midgut cell membrane, the R18 probes would diffuse into the columnar cell membrane and result in a fusion-associated fluorescence increase [46,47]. Based on this, we performed fluorescence-dequenching assays to quantify levels of ODV_R_ attachment and fusion in the midgut of WT and Δ*BmSUH* larvae. As shown in **Fig 7**, maximum levels of R18 were achieved within the first 30 min after inoculation, and the levels remained constant for at least 90 min after inoculation. In the WT group, the amount of bound ODV_R_ ranged between 0.018 ± 0.009 μg and 0.024 ± 0.007 μg; of these, approximately 55% fused (0.009 ± 0.005 μg to 0.014 ± 0.004 μg) (**Fig 7**). By comparison, the binding and fusion levels of ODV_R_ in *BmSUH* mutants, decreased to 57% and 52% of WT, respectively (**Fig 7**). These results show that BmSUH is important for the process of ODV binding and fusion with host midgut epithelial cells.

## Discussion

The interaction between viral attachment proteins and host cellular factors is the first and decisive step in initiating virus entry into the host cells and establishing successful infections [48]. The host proteins that facilitate ODV entry remain largely unknown. Here, we found that the midgut microvilli protein BmSUH appeared to be crucial for efficient invasion of BmNPV. BmNPV “hijacks” BmSUH to facilitate its oral infection by interacting with PIFs to promote ODV virion entry. These findings advance knowledge of the molecular mechanisms and cellular requirements of baculovirus invasion entry into the host midgut and provide a potential new strategy for preventing BmNPV infection.

BmSUH exhibited a distinctly different localization compared to a well-known sucrose-hydrolyzing enzyme, BmSUC1, which is localized in the goblet cell cavities, but a similar pattern to another membrane-bound alkaline phosphatase (mALP), whose location has been demonstrated in the apical microvilli of columnar cells [49,50]. In addition, BmSUH contains an *N*-terminal hydrophobic transmembrane region that may anchor BmSUH in the midgut microvillar membranes, which is the first area of directed contact between BmNPV and host cells after the peritrophic membrane has been penetrated. The *BmSUH* mutants developed into adulthood well without noticeable developmental problems, except a slightly delayed growth in the 5th instar compared to the control under standard feeding conditions, suggesting that BmSUH is dispensable for silkworm development but is beneficial for silkworm growth.

*BmSUH* mutants exhibit higher resistance to BmNPV *per os* infection. When the silkworms were infected by intrahemocoelic injection of BVs, which bypassed the midgut, the survival rate and LD50 had no significant differences in *BmSUH* mutants compared with WT. However, the absence of BmSUH significantly improved silkworm survival rates after being orally infected with OBs, suggesting that BmSUH plays an essential role during the BmNPV midgut infection stage of primary infection. We observed that viral assembly proceeds as normal in the midgut of *ΔBmSUH*, despite the fact that the rate of polyhedron assembly was much slower. In addition, the production of BVs and the mRNA level of viral genes were significantly decreased in *ΔBmSUH*. These observations suggested that BmSUH was not involved in the assembling of virions, but was rather required for virus infection efficiency. Taken together, our findings suggest that BmSUH is required for the efficient of primary infection.

Viral structural proteins are essential for viral genome protection and infection initiation but are likely not required for functions such as DNA replication [40]. The binding partners of BmSUH we identified during BmNPV infection were mainly viral structural proteins and binding proteins. These structural proteins include VP39, GP64, P74 (PIF0), Polyhedrin, and VP91 (PIF8), which are all encoded by baculovirus core genes. Among these, GP64, PIF0, and PIF8 were required for BmNPV entry. GP64 is required for BV binding to the cell surface receptors during the process of BV entry, while PIF0 and PIF8 are required for ODV to enter midgut cells [12,43,51]. The absence of BmSUH reduced infectivity of BmNPV via orally infection instead of by BV injecting. This result was consistent with the effect of deleting PIF proteins, which aborted BmNPV oral infection but the infectious remained following intrahemocoelic injection [9]. The GP64 mRNA level was significantly decreased in Δ*BmSUH* after BmNPV infection. It will be interesting to explore whether the lower BV titer in *BmSUH* mutants is related to the interaction of BmSUH and GP64 in the future.

**Fig 8:**
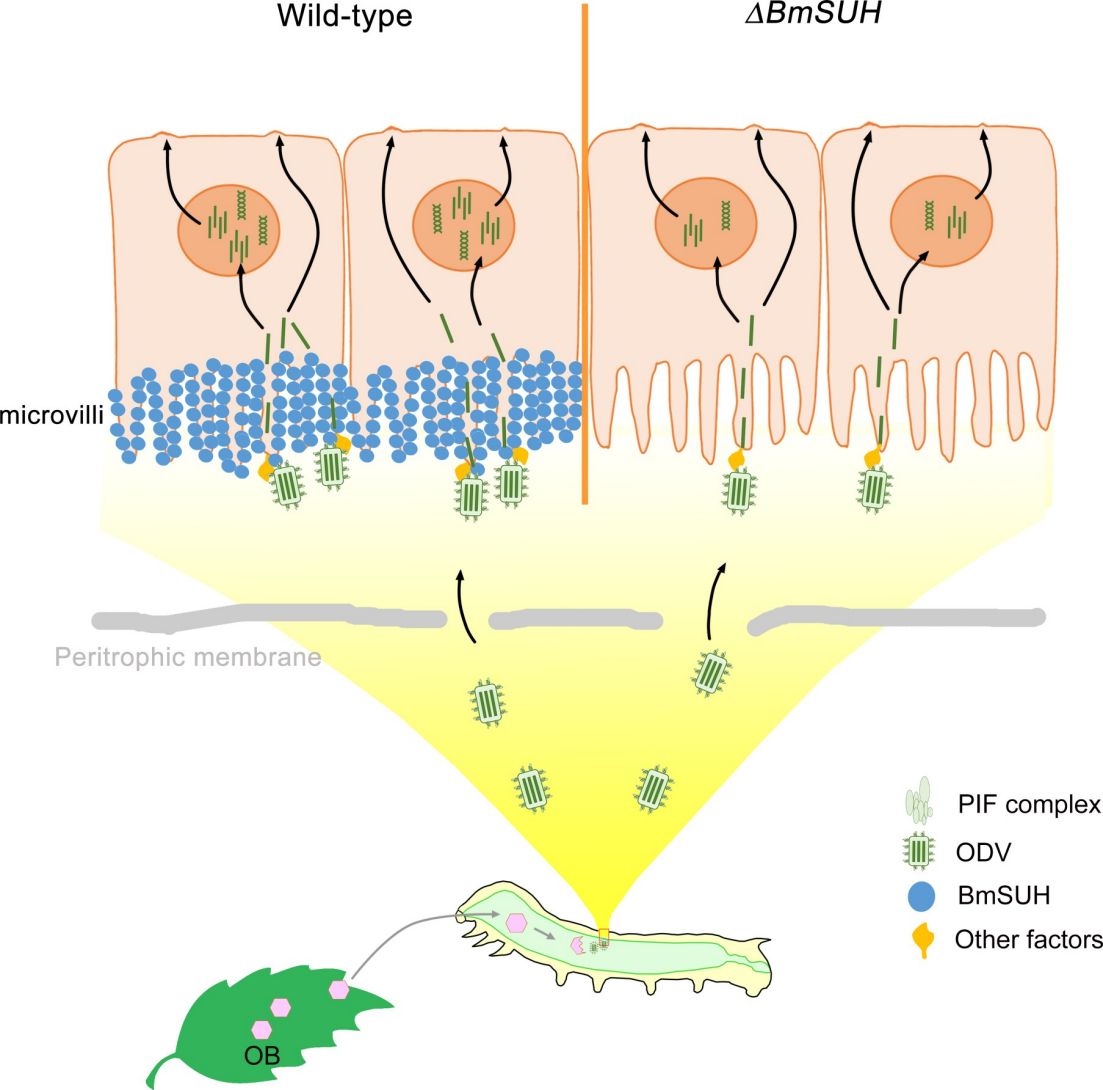
Proposed model of baculovirus oral infection in wild-type or *BmSUH* mutants. BmSUH is widely distributed in the microvilli of *Bombyx mori*. After ODV virions traverse the peritrophic membrane, PIF complex binds to BmSUH and other host factors in the microvilli of epithelial midgut cells. Then the ODV virions fuse with microvilli, releasing nucleocapsids into the cytoplasm. In *BmSUH* mutants, PIF complex could not bind to BmSUH. The loss of BmSUH reduced the ODV entry and led to a lower efficiency of oral infection (the right part). The DNA clipart was obtained from the “Icon Library” of iSlide under a CC0 1.0 Universal license. The silkworm was drawn based on a clipart obtained from the “Icon Library” of iSlide under a CC0 1.0 Universal license. Copyright: https://en.islide.cc/copyright-notice.

PIF-mediated entry is a commonly used and ancient entry mechanism since PIFs are highly conserved in a diverse range of viruses with large, circular, double-stranded DNA genomes that co-evolved with their hosts for millions of years [8,52–54]. BmNPV ODV entry is a complicated process that includes at least 9 PIF proteins. Except PIF5, the other 8 PIFs and Bm91 constitute all the components of a ~500 kDa PIF complex, and this complex is conserved in BmNPV, AcMNPV, and HearNPV [9]. The PIF complex has been found to mediate ODV fusion with the plasma membrane [55]. When deleting PIF0, PIF1 or PIF2, which leads to the disappearance of the PIF complex, the ODV binding and fusing to epithelial cells are blocked (PIF0) or partly blocked (PIF1 or PIF2) [6]. Since BmSUH interacted with PIF complex, conceivably, the PIF complex might potentially mediate the ODV binding process by interacting with BmSUH. In support of this, we found that the amounts of ODV binding and fusion to midgut epithelial cells were reduced in 57% and 52% of WT in the *BmSUH* mutants, respectively. Of note, the loss of BmSUH did not ultimately abolish viral replication or gene expression, showing that the entry process is partly blocked and suggesting that ODV entry is a multistep process that may involve several cell surfaces factors (**Fig 8**). Although it is likely that other host factors mediate in this process, our current findings show that BmSUH acts as a cofactor to enhance ODV binding and fusion to the midgut.

According to the LC-MS/MS and CO-IP results, BmSUH could interact with the PIF complex. However, it seems unlikely that all the components of the PIF complex would directly bind to BmSUH. BmSUH may facilitate BmNPV oral infection in *B*. *mori* midgut via interacting with one or more proteins in the PIF complex. The assembly pattern and structure of PIF complex are still unclear. Therefore, the full characterization of this BmSUH-PIF complex interaction will be of significant importance in the future. It will provide insight into the detailed molecular mechanisms of BmSUH-PIF complex mediated BmNPV entry.

The homologs of SUH have been identified in many lepidopteran species, which implies an evolutionarily conserved role of SUH in Lepidoptera [15–17]. It is worthwhile to determine whether SUH functions as the specific host factor for the BmNPV or as a general target for other NPVs in lepidopteran insects. Clearly, viruses have evolved several different strategies to use the host proteins to their own advantage [56–59]. BmSUH appears as an attractive and potential BmNPV target, for its wide distribution on the midgut epithelial microvilli. In this study, we did not observe visible damage of the midgut epithelium in *BmSUH* mutants. Even so, we cannot rule out the possibility that disruption of BmSUH may reduce BmNPV infectivity through other potential indirect ways. Although our results reveal BmSUH as an important host factor for the initial steps in BmNPV infection, the detailed mechanism of how BmSUH binds to these PIFs to assist the entry of ODV needs further exploration. In addition, this study provides a proof-of-principle for an antiviral strategy—namely, genome editing of a host protein to inhibit the interaction between ODVs and insect midgut epithelial cells required for baculovirus infection.

## Materials and methods

### Insects, cell culture, and the preparation of viral stocks

A multivoltine silkworm strain, Nistari, was maintained at our laboratory and used in this study. BmN cells were maintained in Sf-900 II SFM (Invitrogen) supplemented with 10% fetal bovine serum (Invitrogen) at 27°C as previously described [44]. The WT BmNPV is preserved in our laboratory. BmBacmid was extracted from *E*. *coli* strain BW25113 and used as a template. The OBs were purified from homogenized BmNPV-infected cadavers by differential centrifugation and stored at 4°C for use.

### Antibodies

The polyclonal antibodies against PIF1, PIF2 and PIF3 were gifts from Professor Zhihong Hu (Wuhan Institute of Virology, PR China) [9]. Anti-PIF0 antibody was preserved by our laboratory [60]. The anti-Flag and anti-His antibodies were purchased from HUABIO.

The anti-BmSUH antibody was prepared in our library as follows: A selected sequence (124 aa) of *BmSUH* was amplified by PCR with the primers shown in **S2 Table**, using cDNA of the midgut as a template. PCR products were inserted into the *Bam*H I and *Xho* I sites of the prokaryotic expression vector pCold I (TaKaRa). The recombinant plasmid pCold I-BmSUH was transformed into *E*. *coli* strain BL21. The truncated BmSUH protein was purified and its antiserum was raised in rabbits.

### Construction of CRISPR/Cas9 system in *B*. *mori*

The Cas9 mRNA with T7 promoter was synthesized *in vitro* using a linearized plasmid as a template, following the manufacturer’s instructions of the mMessage mMachine T7 Kit (Ambion). A Poly(A) tailing kit (Ambion) was used to add the Poly(A) signals at the 3′ ends of the mRNAs. The sgRNAs were customized using the online tool of ZiFit Targeter (http://bindr.gdcb.iastate.edu/ZiFiT/) [61]; the sequences of designed sgRNAs are listed in **S2 Table**. The sgRNAs were prepared by transcription performed using T7 MEGAscript Kit (Ambion). Fertilized eggs within 6 hours of oviposition were microinjected with a mixture of Cas9-coding mRNA (300 ng/μl) and *BmSUH* sgRNA (100 ng/μl) [62]. The injected eggs were incubated at 25°C for 7–8 days until hatching, and the newly hatched larvae were reared with fresh mulberry leaves.

### Cas9/sgRNA-mediated mutation screens

To test Cas9/sgRNA-mediated gene alteration, sequencing and western blot were performed. Rearing the G0 generation until adulthood, the total DNA from the moth’s leg was exacted and then used the PCR production for sequencing with the primer pairs (**S2 Table**). When the multiple sequence peaks were shown at the lower position in panel reports, representative DNA sequencing results of the PCR products from G0 moth, indicated that mutation had reduced [63]. The putative mutants of G0 adults were then crossed with WT moths to produce G1 offspring. To investigate mutations in G0 that have been germline transmitted to the G1 offspring, we sequenced the G1 generation in the same way as described previously. In order to quickly acquire homozygous, mosaic individuals for the same loci were crossed with each other [64,65]. In the meantime, the midgut was dissected, ground, lysed, and subjected to western blot using the antiserum against BmSUH. The homozygous mutant was produced when the sequence peaks were single and the signal could not be detected by western blot.

### Immunofluorescence

To investigate the specific expression pattern and localization of BmSUH, the midgut samples of WT and *BmSUH* mutant larvae at L5D3 were dissected, fixed in the paraformaldehyde (PFA) fixative, dehydrated with gradient ethanol, and infiltrated with ethanol and xylene, followed by embedding in paraffin blocks. Subsequently, the 3 μm sections were obtained and deparaffinized with xylene, followed by rehydration with graded methanol series. After heat treatment in citrate buffer, the sections were blocked using goat serum (1:100 dilution) before incubation with the BmSUH antiserum (1:200 dilution) at 4°C overnight. Subsequently, the sections were washed with PBS and incubated with goat anti-rabbit IgG conjugated to fluorescein isothiocyanate (FITC) (1:500 dilution). Finally, the labeled sections were stained with 4’,6’-diamidino-2-phenylindole (DAPI) and examined using a fluorescence microscope (NISELEMENTS; Nikon).

The Open Reading Frame of BmSUH was cloned into the PIZ/V5-His vector. The verified recombinant plasmid was extracted and marked as PIZ-BmSUH. Then the positive constructs were transfected into BmN cells using Cellfectin II (Invitrogen). After 72 h, the medium was discarded and the cells were washed twice with cold PBS. Fix cells with 4% formaldehyde for 15 minutes at 37°C. After washing cells three times in cold PBS, the cells were incubated with 1 mL 5.0 μg/mL WGA conjugate for 10 minutes at room temperature. The cells were washed three times in cold PBS. The following procedures were the same as the immunofluorescence staining mentioned above. Finally, the cells were observed under a Zeiss LSM780 laser-scanning confocal microscope.

### Larval bioassays

To determine the LC50 and LD50_,_ for one set, the newly molted fourth-instar larvae of WT and BmSUH mutants were starved for 12 h and then fed with 10 μL different concentrations of BmNPV (1×10^3^ to 1×10^8^ of OB/mL). For the other set, the LD50 was determined in fifth-instar WT and BmSUH mutant silkworms by haemocoelic injection with different doses of BV diluted in Sf-900 II SFM medium. Each group contained 30 silkworm larvae, and three independent experiments were carried out as biological replicates. Probit analysis of mortality data was conducted using SPSS v.26.0 software (IBM).

For LT50, newly molted fourth-instar larvae including *BmSUH* mutants and WT were starved for 12 h. Each larva was fed with a 1 cm^2^ piece of mulberry leaf smeared with 10 μL OBs at the concentration of 3.48×10^8^ OB/mL. Only larvae that consumed the entire leaf piece were maintained and were reared individually with fresh mulberry leaves. Virus-induced mortality was recorded every 12 h until insect death or pupation. Bioassays with 30 larvae were performed in triplicates. The Kaplan-Meier estimator was applied to evaluate the LT_50_ value using SPSS v.26.0 software (IBM).

To measure the BV production, the hemolymph from fourth instar silkworms that were infected with 10 μL BmNPV at the concentration of 1×10^8^ OB/mL was collected at 48, 72 and 96 hpi. The hemolymph was filtered with 0.45 μm filter membrane before infecting BmN cells. Infectious BV titers were determined by the median tissue culture infectious dose (TCID_50_) end point dilution assay as described [36].

### TEM analyses

To compare the structure of the midgut epithelium of WT and Δ*BmSUH*, the midgut of L5D3 was dissected. To observe the virus in the midgut of WT and Δ*BmSUH*, the fourth-instar *B*. *mori* larvae of WT and Δ*BmSUH* were inoculated orally with 10^6^ OBs. The midgut of each larva was dissected at 96 and 144 hpi. The cropped samples were fixed with 2.5% (v/v) glutaraldehyde overnight at 4°C and ultrathin sections were prepared for TEM (HITACHI H-7650) examination at a 100 kV accelerating voltage.

### Quantitative reverse-transcription PCR

To evaluate the effects of BmSUH protein on viral gene expression, fourth-instar WT and Δ*BmSUH* larvae were inoculated orally with 10^6^ OBs. Total RNA was extracted from WT and Δ*BmSUH* larval midgut using RNAiso Plus (TaKaRa). A PrimeScript RT Reagent Kit with gDNA Eraser (TaKaRa) was used to synthesize cDNA from the extracted RNA. Quantitative mRNA measurements were performed using the TB Green Premix Ex Taq (TaKaRa) on an ABI 7300 Real-Time PCR System (Applied Biosystems). The primer sequences are listed in **S2 Table**. The data were normalized to *B*. *mori* ribosomal protein 49 (Bmrp49) and analyzed with GraphPad Prism v.7.0 with the 2^−ΔΔCt^ method. Two-tailed Student *t* test was used to determine significant differences between different groups of qPCR data. All data were shown as mean ± SEM (N = 3). All assays were carried out in triplicates.

### The generation of recombinant baculoviruses expressing BmSUH and PIFs

Complete DNA encoding *BmSUH*, nine *PIFs* and *Bm91* were amplified with specific primers (**S2 Table**) using the cDNA of the *B*. *mori* midgut and BmBacmid as templates, respectively. PCR products were subcloned into pFastBac1 donor plasmid. The recombinant bacmids were constructed according to the protocol of Bac-to-Bac baculovirus expression system (Invitrogen). The recombinant bacmids were transfected into BmN cells using Lipofectamine 3000 Transfection Kit (Invitrogen) to generate recombinant baculovirus. His-tagged BmSUH (BmSUH-His) and Flag-tagged PIFs (PIFs-Flag) were expressed in baculovirus-transformed BmN cells at 27°C and harvested after four days’ post-infection. The BmSUH-His and PIF-Flag containing viral supernatants were also collected and stored at 4°C for use in the co-immunoprecipitation assay.

### Co-immunoprecipitation assay and western blot analysis

To prepared the samples for LC-MS/MS analysis, the CO-IP were performed by using midguts tissue from virus infected and uninfected larvae. The midgut tissues were lysed in 1 ml of IP buffer [20mM Tris (pH7.5), 150mM NaCl, 1% Triton X-100, 1mM PMSF] (Beyotime, Biotechnology). Lysates were centrifuged at 12,000 × g for 5 minutes at 4°C. One-tenth volumes of the supernatants were preserved as the input controls, and the rest lysates incubated at 4°C overnight with anti-BmSUH antibody. Protein A+G agarose (Beyotime Biotechnology) were added to the lysate solution for the last 4 h of incubation. At last, the agaroses were washed four times with the IP lysis buffer and then boiled in 1× SDS sample buffer containing β-mercaptoethanol.

To confirm the interaction of PIF proteins with BmSUH, we carried out two sets of in vitro CO-IP in BmN cells. The BmN cells were infected or co-infected by the BmSUH-His and PIF-Flag viruses at an M.O.I. of 5, after which infected cells were collected at 72 hpi and washed twice with 1×PBS. The collected cells were lysed in Western and IP buffer as described above. For one set of lysates, the CO-IP was performed using protein A+G agarose as described above. Western blot analyses were conducted after separation by SDS-polyacrylamide gel electrophoresis (PAGE) and transfer to a PVDF membrane. The primary antibodies used were anti-P74 (1:500 dilution), anti-PIF1 (1: 2,000 dilution), anti-and anti-PIF2 (1:2,000 dilution) and anti-Flag antibody (1:5,000 dilution).

Another set of lysates were incubated with anti-Flag Immunomagnetic Beads (Bimake) at 4°C overnight. After that, the beads were boiled in 1× SDS sample buffer and subjected to SDS- PAGE and examined by western blot. anti-Flag antibody to detect the expression and immunoprecipitation of the Flag-tagged proteins and anti-His antibody to determine if BmSUH was co-immunoprecipitated with the PIFs.

### LC-MS/MS analysis

The IP protein samples were separated by SDS-PAGE until the dye front reached the bottom of the gel. After staining with Coomassie Brilliant Blue the 70kDa and 100kDa bands were excised and subjected to LC-MS/MS analysis by Q Exactive mass spectrometer. The experiment and data analysis were supported by Shanghai Applied Protein Technology.

GO analysis was performed on the proteins identified by LC-MS/MS. The R package "TopGO" was used to analyze the GO enrichment, and the R package "ggplot" was used to visualize the results.

### Fluorescence-dequenching assay

To determine the role of *BmSUH* in viral invasion of midgut epithelial cells, fluorescence-dequenching assays were performed. To prepare ODVs, supernatants of OB lysates were purified by ultracentrifugation through 30%−60% (w/v) sucrose as previously described [60]. Subsequently, the purified ODV was labeled with the self-quenching fluorescent probe octadecyl rhodamine B chloride (R18; Invitrogen Life Technologies), as described previously [10]. Labeled ODV_R_ was quantified using BCA protein Assay Kit (Takara) and stored in the dark at 4°C until use. Newly molted fourth-instar WT and Δ*BmSUH* larvae were inoculated orally with 3 μg labeled ODV_R_ (n = 24 larvae) or PBS (n = 24 larvae). At 30-, 60-, and 90-min post-inoculation, after which the midgut of each larva was dissected and rinsed in 200 μL chilled separation buffer (100 mM NaCO_3_, 100 mM KCl, 100 mM EGTA, pH 9.5) and stored in the dark at −80°C until use. The amounts of ODV bound and fused were measured using FLUOROMAX-4 fluorescence spectrophotometer (Horiba Jobin Yvon) at excitation/emission wavelengths of 560nm/583nm, after which the relative fluorescence units per mg ODV_R_ protein was calculated.

## Supporting information

S1 Fig The sequence results of WT and homozygote mutant silkworm. The target site location was shown in green and PAM sequences were shown in red. The black underlines highlight the target site location and the nearby sequences. (A) The chromatograms of WT. (B) Representative chromatograms of homozygotes. The deletion sequences were shown in gray, and the cutting site was indicated by a red arrow. (C) The mutation events were confirmed by sequencing.
(TIF)

S2 Fig The *BmSUH* mutants have no significant defects in fecundity and viability. (A) Images of the eggs produced by WT and *BmSUH* mutants (n = 20 couples per group). (B) The number of eggs (left panel) and the hatching rate of WT and Δ*BmSUH* (right panel). Data were presented as mean ± SD and analyzed by SPSS v.26.0 software using two-tailed Student *t* test. ns, P > 0.05.
(TIF)

S3 Fig TEM observation of midgut columnar cells in wild-type and *BmSUH* mutants. Silkworm specimens were obtained from day 3 of the 5th instar (L5D3) larval. N: nucleus, M: microvilli. The white triangle indicates mitochondria, and the black arrow indicates the microapocrine vesicles.
(TIF)

S4 Fig BmSUH was responsive to BmNPV. (A) Western blot analysis of BmSUH protein level in *B*. *mori* before and after BmNPV oral infection. Anti-tubulin was used as an internal control. (B) Quantification of western signal as the mean ±SD (n = 3 biological replicates). Signals were quantitated using Image J software. **p < 0.01 and ***p < 0.001 by two-tailed Student *t* test.
(TIF)

S5 Fig The GO enrichment analysis of the binding partners of BmSUH. Midgut proteins of 100 kDa gel which appeared after BmNPV infection were detected by LC-MS/MS. The identified genes were submitted to GO enrichment analysis. BP: Biological process, CC: Cellular component, MF: Molecular function.
(TIF)

S1 Table Viral proteins detected by LC-MS/MS following IP using anti-BmSUH. (XLSX)

S2 Table The primers used in this study. (XLSX)
