# Supplementary figures and images for: Midgut membrane protein BmSUH facilitates Bombyx mori nucleopolyhedrovirus oral infection
Source: PLoS Pathog. 2022 Nov 16;18(11):e1010938. doi: 10.1371/journal.ppat.1010938 (PMC9668127; doi:10.1371/journal.ppat.1010938)

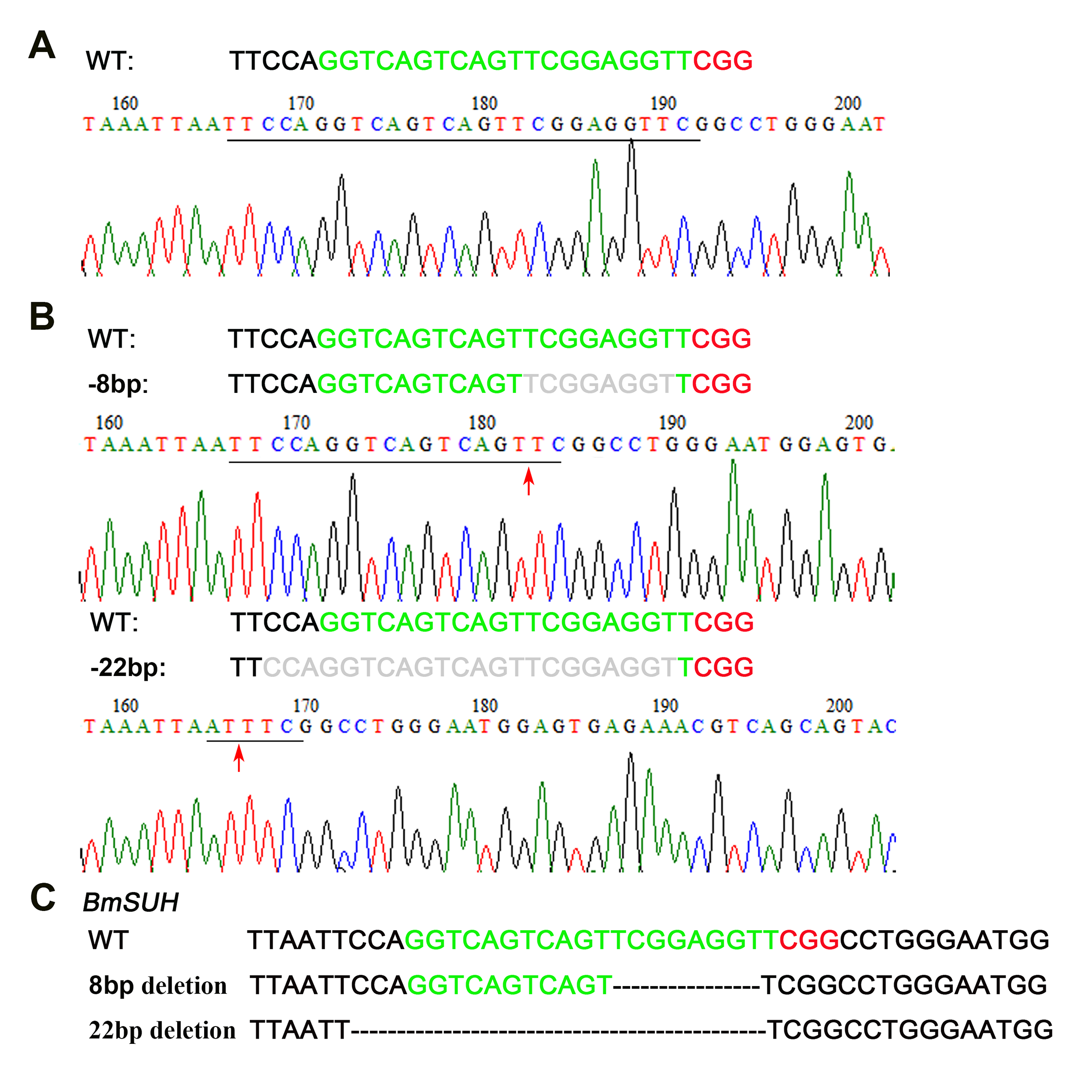

Supplement: S1 Fig — The target site location was shown in green and PAM sequences were shown in red. The black underlines highlight the target site location and the nearby sequences. (A) The chromatograms of WT. (B) Representative chromatograms of homozygotes. The deletion sequences were shown in gray, and the cutting site was indicated by a red arrow. (C) The mutation events were confirmed by sequencing. (TIF) [file ppat.1010938.s001.tif]

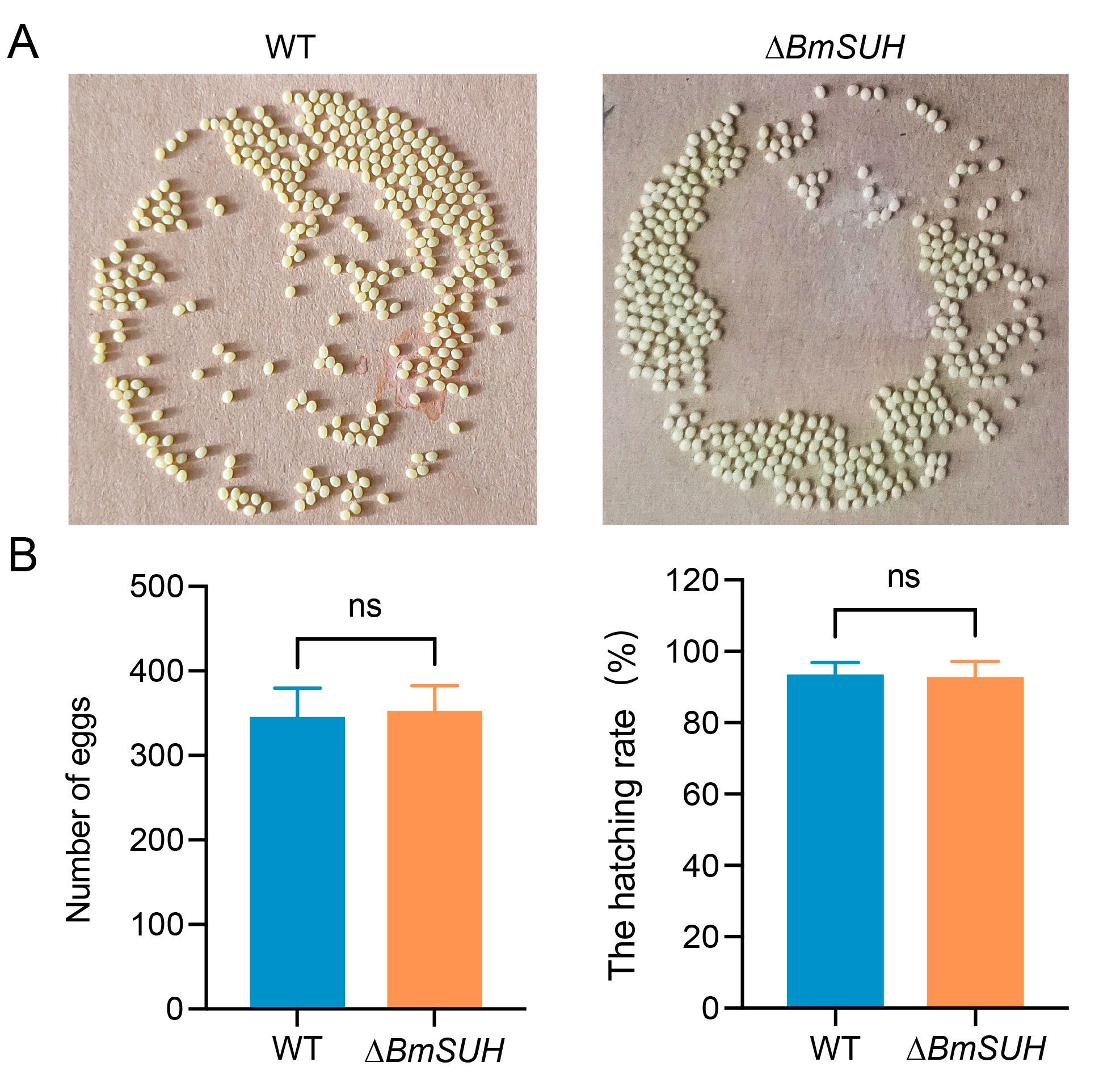

Supplement: S2 Fig — (A) Images of the eggs produced by WT and BmSUH mutants (n = 20 couples per group). (B) The number of eggs (left panel) and the hatching rate of WT and ΔBmSUH (right panel). Data were presented as mean ± SD and analyzed by SPSS v.26.0 software using two-tailed Student t test. ns, P > 0.05. (TIF) [file ppat.1010938.s002.tif]

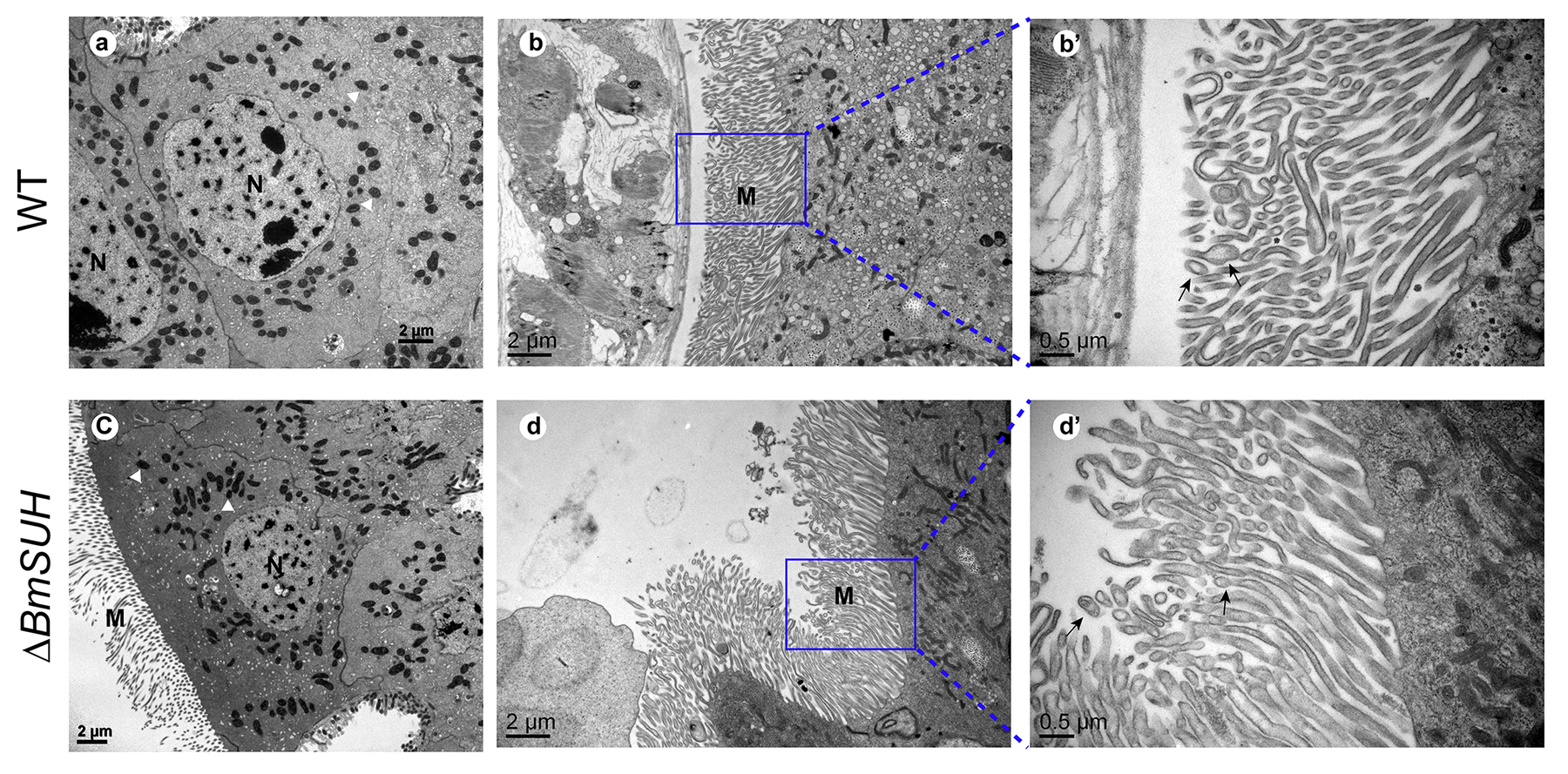

Supplement: S3 Fig — Silkworm specimens were obtained from day 3 of the 5th instar (L5D3) larval. N: nucleus, M: microvilli. The white triangle indicates mitochondria, and the black arrow indicates the microapocrine vesicles. (TIF) [file ppat.1010938.s003.tif]

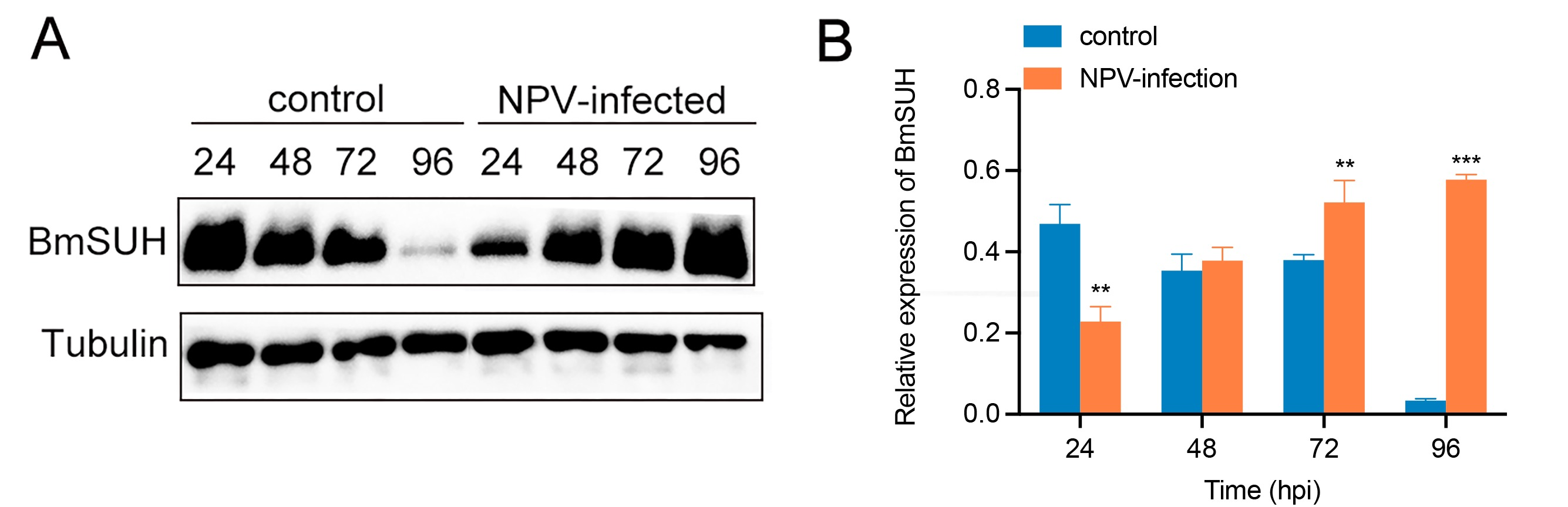

Supplement: S4 Fig — (A) Western blot analysis of BmSUH protein level in B. mori before and after BmNPV oral infection. Anti-tubulin was used as an internal control. (B) Quantification of western signal as the mean ±SD (n = 3 biological replicates). Signals were quantitated using Image J software. **p < 0.01 and ***p < 0.001 by two-tailed Student t test. (TIF) [file ppat.1010938.s004.tif]

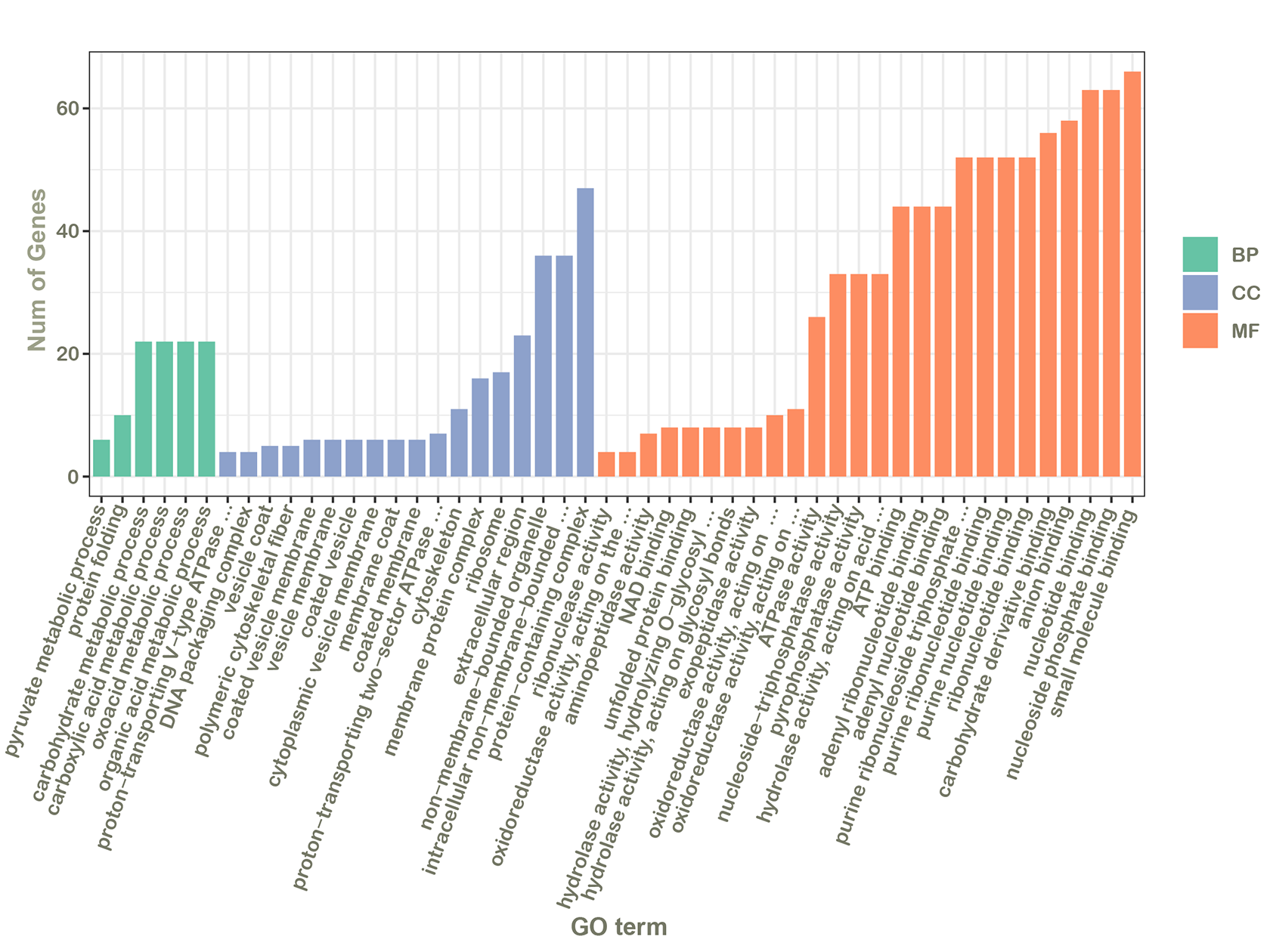

Supplement: S5 Fig — Midgut proteins of 100 kDa gel which appeared after BmNPV infection were detected by LC-MS/MS. The identified genes were submitted to GO enrichment analysis. BP: Biological process, CC: Cellular component, MF: Molecular function. (TIF) [file ppat.1010938.s005.tif]
